# Ajugol's upregulation of TFEB-mediated autophagy alleviates endoplasmic reticulum stress in chondrocytes and retards osteoarthritis progression in a mouse model

**DOI:** 10.1186/s13020-023-00824-7

**Published:** 2023-09-07

**Authors:** Jingtao Wu, Heng Yu, Yangcan Jin, Jingquan Wang, Liwen Zhou, Teng Cheng, Zhao Zhang, Binghao Lin, Jiansen Miao, Zhongke Lin

**Affiliations:** 1 Department of Orthopaedics, Wenzhou Key Laboratory of Perinatal Medicine, The Second Affiliated Hospital and Yuying Children’s Hospital of Wenzhou Medical University, Wenzhou, 325000 Zhejiang Province China; 2 Key Laboratory of Orthopaedics of Zhejiang Province, Wenzhou, 325000 Zhejiang Province China; 3 The Second School of Medicine, Wenzhou Medical University, Wenzhou, 325000 Zhejiang Province China; 4 The First School of Medicine, Wenzhou Medical University, Wenzhou, 325000 Zhejiang Province China

**Keywords:** Osteoarthritis, Ajugol, TBHP, TFEB, Autophagy, ER stress, DMM

## Abstract

**Background:**

Osteoarthritis (OA), a degenerative disease with a high global prevalence, is characterized by the degradation of the extracellular matrix (ECM) and the apoptosis of chondrocytes. Ajugol, a extract derived from the herb Rehmannia glutinosa, has not yet been investigated for its potential in modulating the development of OA.

**Methods:**

We employed techniques such as western blotting, immunofluorescence, immunohistochemistry, X-ray imaging, HE staining, and SO staining to provide biological evidence supporting the role of Ajugol as a potential therapeutic agent for modulating OA. Furthermore, in an in vivo experiment, intra-peritoneal injection of 50 mg/kg Ajugol effectively mitigated the progression of OA following destabilization of the medial meniscus (DMM) surgery.

**Results:**

Our findings revealed that treatment with 50 μM Ajugol activated TFEB-mediated autophagy, alleviating ER stress-induced chondrocyte apoptosis and ECM degradation caused by TBHP. Furthermore, in an in vivo experiment, intra-peritoneal injection of 50 mg/kg Ajugol effectively mitigated the progression of OA following destabilization of the medial meniscus (DMM) surgery.

**Conclusion:**

These results provide compelling biological evidence supporting the role of Ajugol as a potential therapeutic agent for modulating OA by activating autophagy and attenuating ER stress-induced cell death and ECM degradation. The promising in vivo results further suggest the potential of Ajugol as a treatment strategy for OA progression.

**Supplementary Information:**

The online version contains supplementary material available at 10.1186/s13020-023-00824-7.

## Introduction

Osteoarthritis (OA) is a prevalent and debilitating joint disorder that affects millions of individuals worldwide [1]. Environmental factors such as joint trauma, obesity, mechanical stress, and inflammatory mediators play pivotal roles in triggering and exacerbating the disease [2]. The pathological features include cartilage degradation, osteophyte formation, subchondral bone remodeling, synovial inflammation, and alterations in the menisci and ligaments [3].

Among the various cell types in the joint, chondrocytes play a pivotal role in maintaining cartilage homeostasis and contribute significantly to OA pathobiology [4]. Chondrocyte apoptosis is a critical process implicated in the pathogenesis of OA [5, 6]. It contributes to cartilage degradation, triggers inflammatory responses, and influences extracellular matrix (ECM) remodeling [7]. In OA, chondrocytes undergo phenotypic changes characterized by altered matrix synthesis and degradation. Upregulated matrix metalloproteinases (MMPs) and a disintegrin and metalloproteinase with thrombospondin motifs 5(ADAMTS5) contribute to excessive ECM degradation, leading to the loss of proteoglycans and collagen fibers [8, 9].

When cells are subjected to oxidative stress, it leads to endoplasmic reticulum (ER) stress [10]. During ER stress, there is an accumulation of unfolded and misfolded proteins in the ER, triggering a signaling pathway known as the unfolded protein response (UPR), which aims to restore ER homeostasis and alleviate the stress [11]. The UPR involves the activation of three main transmembrane sensors located on the ER membrane: inositol-requiring enzyme 1 (IRE1), protein kinase RNA-like ER kinase (PERK), and activating transcription factor 6 (ATF6) [12–14]. Upon activation, these sensors initiate a cascade of signaling events to address ER stress and restore proper protein folding capacity. Prolonged activation of the UPR can lead to cell death, ultimately impairing tissue function and contributing to disease pathogenesis [15]. Therefore, we speculate that pharmacological interventions targeting endoplasmic reticulum (ER) stress relief may represent a viable and effective approach to inhibit the progression of OA. Considering that tert-butyl hydroperoxide (TBHP) is a stable compound and has been extensively studied in the context of OA, we chose this compound to simulate cellular oxidative stress in our investigation [16–18].

TFEB (Transcription Factor EB) is a master regulator of cellular homeostasis with diverse functions in various cellular processes [19]. It plays a crucial role in maintaining cellular quality control, coordinating lysosomal biogenesis and autophagy, and regulating cellular metabolism [20]. Studies have shown that TFEB mediated autophagy can delay chondrocyte senescence and thus delay the progression of osteoarthritis [21, 22]. An increasing body of research suggests that proper activation of autophagy can alleviate endoplasmic reticulum (ER) stress and promote cell survival under oxidative stress conditions [23, 24]. Therefore, we hypothesize that TFEB-mediated autophagy may alleviate ER stress and delay chondrocyte apoptosis.

Rehmannia glutinosa, a commonly used herb in traditional Chinese and Korean medicine, has been extensively employed for its therapeutic properties in treating a range of conditions such as inflammation [25], diabetes [26], and lipid metabolism disorders [27]. Within the roots of Rehmannia glutinosa, there is an abundant presence of a compound called Ajugol, an iridoid glycoside known for its antioxidant and anti-inflammatory effects. Ajugol has been demonstrated to exert significant effects in non-alcoholic fatty liver disease [27], asthma [28], and neuroinflammation models [29]. However, its role in mitigating the progression of OA has not been reported to date.

In our study, we investigated the impact of Ajugol on endoplasmic reticulum (ER)-induced chondrocyte apoptosis and extracellular matrix (ECM) degradation, and described its potential underlying mechanisms. Additionally, we evaluated the therapeutic efficacy of drug treatment on medial meniscal destabilization (DMM) in a mouse model of OA.

## Materials and methods

### Isolation and culture of murine chondrocytes

A three-day-old lactating mouse was euthanized with an excessive dose of pentobarbital. Its hind legs were then amputated and placed in PBS. Subsequently, the knee joint cartilage was carefully dissected using microscissors and placed in a 2 mg/ml type II collagenase solution for 2 h at 37 degrees Celsius. The cells were then separated using a centrifuge at 1000 revolutions per minute and seeded in a complete culture medium containing DMEM/F12. The culture was maintained in a CO2 concentration of 5% at 37 degrees Celsius. Upon reaching 80% cell confluency, passaging was performed using post-P2 chondrocytes for the next steps.

### SiRNA transfection

TFEB small interfering RNA (siRNA) was obtained from Ribobio (Guangzhou, China). Chondrocytes were transfected with si-TFEB or si-NC in a complete culture medium without antibiotics when the cell density reached 30%. After reaching a cell confluency of > 90% following a 3-day incubation, the cells were seeded in a complete culture medium containing antibiotics for further experiments. The transfection efficiency was assessed using RT-PCR.

### TUNEL staining

The TUNEL assay kit was obtained from Vazyme (Nanjing, China). On the first day, we placed 12-well culture plates in six-well plates and seeded chondrocytes at 80% confluency. On the second day, after the chondrocytes adhered to the plate, we applied the appropriate stimulation to the cells. On the third day, we performed TUNEL staining according to the instructions provided by the kit. Finally, nuclear staining was performed using mounting medium containing DAPI, and the samples were observed under a fluorescence microscope (Nikon ECLIPSE Ti microscope).

### Live-dead cell staining

The Calcein/PI Cell Viability/Cytotoxicity Assay Kit was purchased from Beyotime (Shanghai, China). The working solution was prepared according to the instructions provided by the kit and added to the 24-well plate containing seeded cells. The plate was then incubated at 37 degrees Celsius, protected from light, for 30 min. Finally, the samples were observed under a fluorescence microscope (Nikon ECLIPSE Ti microscope).

### EdU staining

EdU Cell Proliferation Kit with Alexa Fluor 488 was obtained from Beyotime (Shanghai, China). Following the manufacturer's instructions, a 2X working solution was prepared and added to a 24-well plate containing an equal amount of culture medium with stimulated chondrocytes. The working solution was diluted to 1X, and the plate was incubated at 37 °C in a light-protected environment for 4 h. Finally, observations were made under a fluorescence microscope.

### Cell viability assay

Chondrocyte viability was assessed using the CCK-8 assay kit (Vazyme, Nanjing, China). Following the instructions, 80,000 cells/ml were seeded into a 96-well plate. The corresponding treatments were administered on the following day. On the third day, after aspirating the culture medium, DMEM/F12 containing 10% CCK-8 solution was added to the wells. The plate was then incubated at 37 degrees Celsius in a cell culture incubator for 2 h. Subsequently, measurements were taken using a spectrophotometer (Thermo Fisher).

### Western blot

We added 1 mM PMSF RIPA lysis buffer to the culture dish to disrupt the cell membrane. The disrupted cells were then collected in an EP tube using a scraper. The EP tube was subsequently centrifuged at 12,000 rpm to obtain the supernatant. The protein concentration was measured using a BCA assay kit (Vazyme, Nanjing, China). All proteins were prepared to a concentration of 30 μg/20 μl. For protein separation, 20 mg of the protein samples were loaded onto SDS-PAGE gels and then transferred onto membranes (Millipore, USA). After incubating the membranes with skimmed milk for 2 h, they were mixed with the antibodies. The membranes were incubated with the primary antibodies at 4 °C for 12 h. Subsequently, the bands were incubated with the respective secondary antibodies at room temperature. The density of each band was detected using the Tanon Image 4600 system, and the results were quantified using ImageJ software.

ROS Assay: The ROS assay kit was obtained from Beyotime, and DCFH-DA was diluted in serum-free culture medium at a ratio of 1:1000 as per the manufacturer's instructions, resulting in a final concentration of 10 μmol/L. Cell culture medium was removed from the six-well plate, and 1 mL of the diluted DCFH-DA was added. Incubation was carried out at 37 °C in a cell culture incubator for 20 min. Cells were washed three times with serum-free cell culture medium to thoroughly remove any residual DCFH-DA that had not entered the cells. Subsequently, cells were stained with Hoechst 33,342 live cell staining solution for nuclear staining, following the manufacturer's instructions. Finally, observations were made under a Leica inverted microscope.

### Toluidine blue staining

The Toluidine Blue staining reagent was procured from servicebio. Initially, 10,000 cells were seeded per well in a 96-well plate. After three days, the cells were subjected to the respective stimulus for 24 h. Subsequently, the liquid in the 96-well plate was aspirated, and the cells were fixed with 4% paraformaldehyde. Then, 100 μl of Toluidine Blue staining reagent was added and allowed to incubate for 30 min. Afterward, the cells were washed with PBS for 5 min × 5 times and observed under an inverted microscope.

### Immunofluorescence staining

The cells were subjected to a treatment with 0.1% Triton-X for 5 min, followed by blocking with 10% goat serum at 37 °C for 30 min. Subsequently, the samples were incubated with antibodies targeting Bag3 (1:200), LC3 (1:150), and lamp1 (1:200). After that, chondrocytes were further incubated with secondary antibodies for 30 min. To visualize the cell nuclei, DAPI staining was performed for 5 min. The samples were then examined under fluorescence microscopy. The fluorescence intensity of the samples was quantified using Image J software (Bethesda, Maryland, USA).

### Molecular docking

Molecular Interaction between TFEB and Ajugol. In this study, the impact of Ajugol on TFEB activation was investigated through computer MD analysis. It was found that the binding affinity was −2.23 kcal mol-1, and Ajugol exhibited excellent embedding within the structural domain interacting with TFEB. Local interaction maps indicated the formation of hydrogen bonds between Ajugol and the residues ASN-174 and MET-177. These results suggest a strong affinity between Ajugol and the TFEB complex protein.

### OA model

In compliance with ethical review and statistical analysis requirements, we obtained 30 male C57BL/6 mice, aged 10 weeks, from the Shanghai Animal Center of the Chinese Academy of Sciences. The mice were randomly divided into five groups: Sham, DMM, DMM + Low Ajugol, DMM + High Ajugol, and DMM + Indometacin.

Ajugol and Indometacin were purchased from MCE. The osteoarthritis (OA) model was established using the DMM (Destabilization of the Medial Meniscus) technique [30]. In the DMM + Low Ajugol group, mice received daily intraperitoneal injections of Ajugol dissolved in DMSO at a dose of 25 mg/kg, while the DMM + High Ajugol group received a dose of 50 mg/kg [27]. As a control, mice in the DMM + Indometacin group received intraperitoneal injections of Indometacin at a dose of 3 mg/kg [31]. The Sham surgery and DMM groups were administered an equivalent volume of DMSO. At the end of the 8th week of treatment initiation, mice were euthanized, and tissues were collected for biochemical and histological analysis. The animals were housed in a well-ventilated environment with a temperature maintained between 24 and 26 °C. A 12-h light–dark cycle was provided, ensuring adequate lighting. Ample water and food were provided for the mice.

### X-ray imaging analysis

After 8 weeks after DMM, we sacrificed mice with an overdose of pentobarbital. Then we carefully cut off the mouse's hind legs, remove soft tissues such as muscle skin, and then arrange the tibia and femur at an angle of 120°. This is followed by direct X-rays. X-ray imaging analysis was conducted using an Xpert 80 system (Kubtec, KUB Technologies Inc.) at 50 kV and 160 μA to assess the extent of cartilage damage and calcification.

### Histopathological analysis

Following euthanasia, tissues were fixed in 4% paraformaldehyde for 24 h, followed by decalcification in pH 7.4 EDTA decalcification solution. Subsequently, the tissues were dehydrated and embedded in paraffin. Once decalcification was completed, tissue se ctions of 5 μm thickness were obtained using a microtome. Subsequent to sectioning, HE (hematoxylin–eosin) staining and SO (safranin O) staining were performed according to the manufacturer's instructions. The severity of osteoarthritis (OA) was assessed using the International Osteoarthritis Research Society (OARSI) histopathology scoring system. Histopathological images of the tissues were acquired using a digital slide scanning system (OLYMPUS) and evaluated by two independent investigators blinded to the treatment conditions.

### Immunohistochemical

Following routine deparaffinization and hydration, the tissue sections were washed in PBS for 5 min, repeated for three cycles. Then, the sections were treated with an endogenous peroxidase inhibitor (ZSBio, Beijing, China) for 10 min, followed by three washes with PBS. Subsequently, the sections were incubated with gastric protease (ZSBio, Beijing, China) at 37 °C for 30 min. Blocking of the tissue sections was performed using 10% (v/v) goat serum albumin at 37 °C for 30 min. The sections were then incubated overnight at 4 °C with specific primary antibodies (diluted 1:100) targeting Collagen II, TFEB, ATF4, and CHOP. On the following day, the sections were incubated with a horseradish peroxidase-conjugated goat anti-rabbit IgG polymer (ZSBio, Beijing, China) for 1 h, followed by DAB staining. Similarly, histopathological images of the tissues were acquired using a digital slide scanning system (OLYMPUS) and evaluated using the aforementioned methods.

### Statistical analysis

The experiments were replicated a minimum of three times. The data are presented as the mean ± standard deviation. Statistical analyses were performed using SPSS 23.0 software. One-way analysis of variance (ANOVA) and Tukey's test were used to compare cells and tissues subjected to different treatments. Nonparametric data (OARSI scores) were analyzed using the Kruskal–Wallis H test. A p-value less than 0.05 was considered statistically significant. *P < 0.05, ** *P* < 0.01, ***P < 0.001.

## Result

### Ajugol toxicity, and effects on chondrocyte activity

**Fig. 1:**
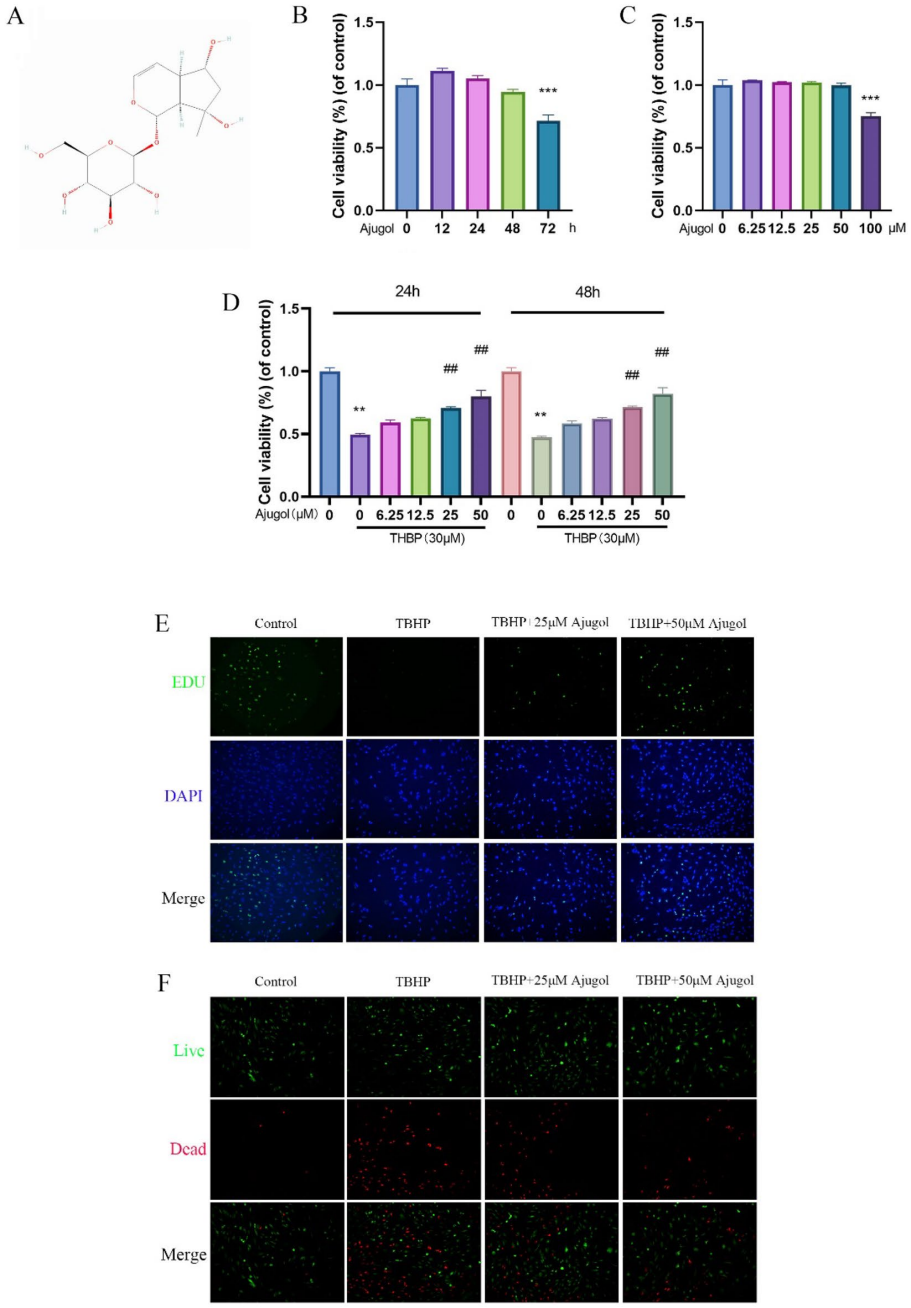
Ajugol toxicity, and effects on chondrocyte activity. **A** Chemical structure of Ajugol. **B** CCK8 assay results illustrating the viability of chondrocytes treated with various concentrations of Ajugol for 24 h. **C** Chondrocyte viability after treatment with Ajugol for 12, 24, 48 and 72 h, as detected by CCK8 assay. **D** Cell viability of TBHP stimulated (30 μM) chondrocytes after treatment with varying concentrations of Ajugol. **E** EdU staining of TBHP stimulated (30 μM) chondrocytes after treatment with varying concentrations of Ajugol. **F** Live-dead staining of TBHP stimulated (30 μM) chondrocytes after treatment with varying concentrations of Ajugol. All values are presented as mean ± standard deviation (n = 3). *P < 0.05,**P < 0.01,***P < 0.001

To investigate the optimal concentration and duration of Ajugol treatment on chondrocytes, as well as its protective effects on these cells, we conducted CCK-8 experiments. We found that Ajugol concentrations equal to or below 50 μM had no significant effect on cell viability, while a concentration of 100 μM inhibited chondrocyte activity (Fig. 1B). Similarly, stimulation of chondrocytes with 50 μM Ajugol for 72 h resulted in suppressed cell viability (Fig. 1C). Furthermore, we examined the cytoprotective effects of different concentrations and exposure times of Ajugol on chondrocytes treated with TBHP. To simulate oxidative stress in chondrocytes, we used 30 μM TBHP for 24 h and subsequently administered various concentrations of Ajugol [32]. CCK-8 assays were performed after 24 and 48 h. Our findings demonstrated that Ajugol mitigated oxidative stress in chondrocytes, with the optimal protective effect observed at a concentration of 50 μM. Additionally, it appeared that a 24-h treatment with Ajugol provided greater protection compared to 48 h. To further confirm the protective effects of Ajugol on chondrocytes, we conducted EdU staining and Live-Dead Cell Staining. These experiments revealed that Ajugol rescued TBHP-induced proliferation limitations and chondrocyte death (Fig. 1D, E). Taken together, these results demonstrate that Ajugol can protect chondrocytes from oxidative stress induced by TBHP.

### Ajugol alleviates TBHP-induced apoptosis, ECM degradation and ROS accumulation in chondrocytes

**Fig. 2:**
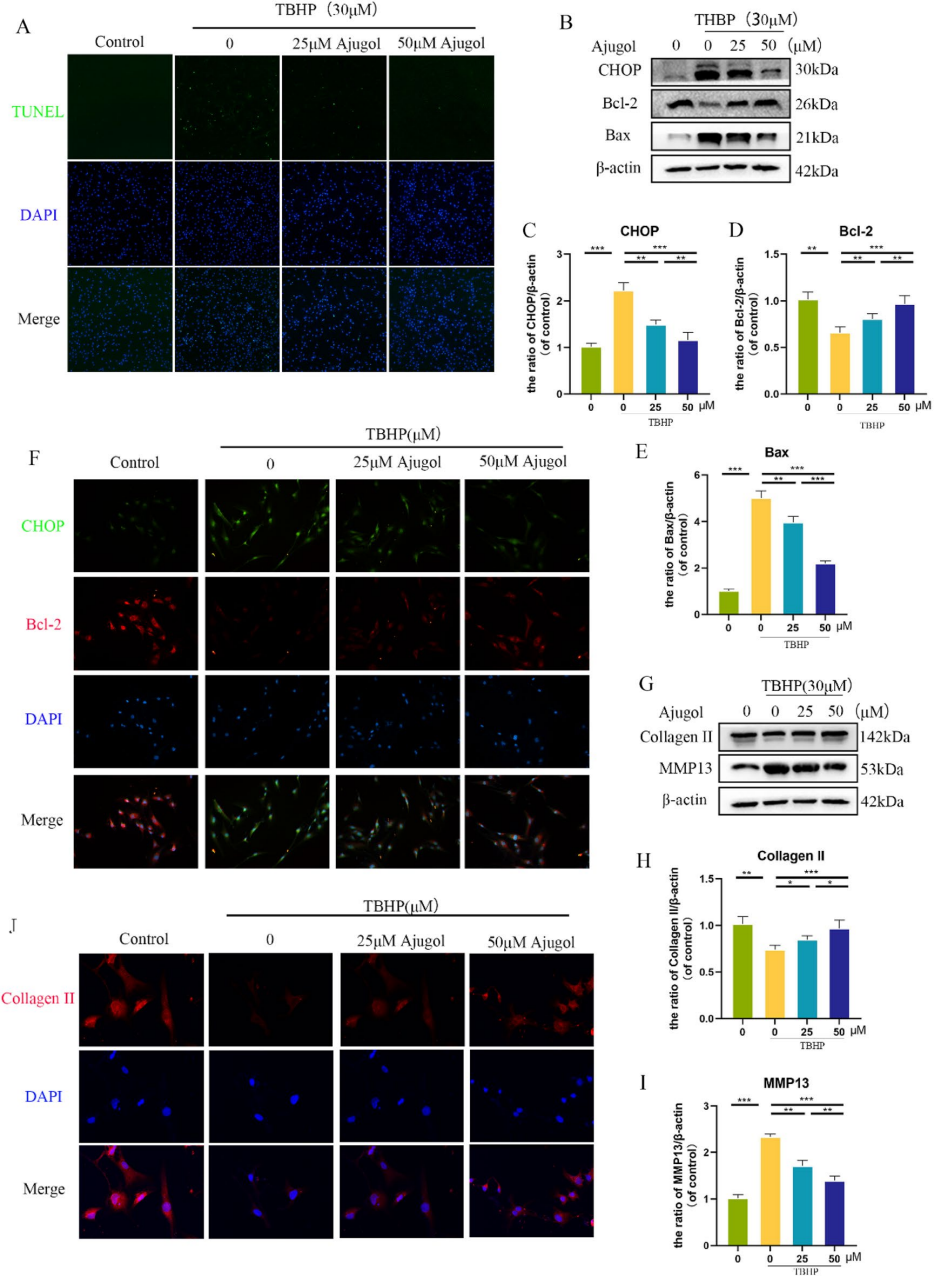
Ajugol alleviates TBHP-induced apoptosis and ECM degradation in chondrocytes. **A** TUNEL staining images showing the effect of different gradients of Ajugol following the addition of 30 μM TBHP. **B**–**E** Protein expression levels of CHOP, Bcl-2, and Bax in hondrocytes stimulated with 30 μM TBHP and treated with different concentration gradients of Ajugol. **F** Immunofluorescence results of CHOP and Bcl-2 in chondrocytes stimulated with 30 μM TBHP and treated with different concentration gradients of Ajugol. **G**–**I** Protein levels of Collagen II and MMP13 in each group; **J** Immunofluorescence staining of Collagen II. All values are presented as mean ± standard deviation (n = 3). *P < 0.05,** *P* < 0.01,***P < 0.001

To investigate whether Ajugol can mitigate TBHP-induced apoptosis, we initially performed TUNEL staining and observed a significant enhancement of its anti-apoptotic effect with increasing Ajugol concentrations (Fig. 2A). To further validate our findings, we conducted Western blotting (WB). Bax (Bcl-2-associated X protein) and Bcl-2 are commonly used proteins to assess mitochondrial apoptosis, while CHOP (C/EBP Homologous Protein) is an effector protein of endoplasmic reticulum-induced apoptosis. The results demonstrated a significant decrease in pro-apoptotic Bax and CHOP expression under Ajugol intervention, accompanied by an elevation in the anti-apoptotic protein Bcl-2(Fig. 2B–E). Immunofluorescence staining yielded similar results (Fig. 2F). Subsequently, we explored whether Ajugol could attenuate TBHP-induced ECM degradation. Similarly, we conducted WB and immunofluorescence staining and observed a significant increase in Collagen II expression, indicative of ECM protection, in the presence of Ajugol. Conversely, MMP13, an enzyme associated with ECM degradation, displayed a reversed trend (Fig. 2G–J). Furthermore, we conducted toluidine blue staining directly on chondrocytes to assess the degradation and synthesis of ECM. As illustrated in Additional file 1: Figure S1A, Ajugol partially reversed the ECM degradation induced by TBHP. We then examined the ROS accumulation in chondrocytes using ROS detection kit. It was found that Ajugol alleviated TBHP-mediated ROS accumulation (Additional file 1: Figure S2A). These findings collectively suggest that Ajugol can protect chondrocytes from TBHP-induced apoptosis and ECM degradation.

### Ajugol alleviates TBHP-induced endoplasmic reticulum (ER) stress

**Fig. 3:**
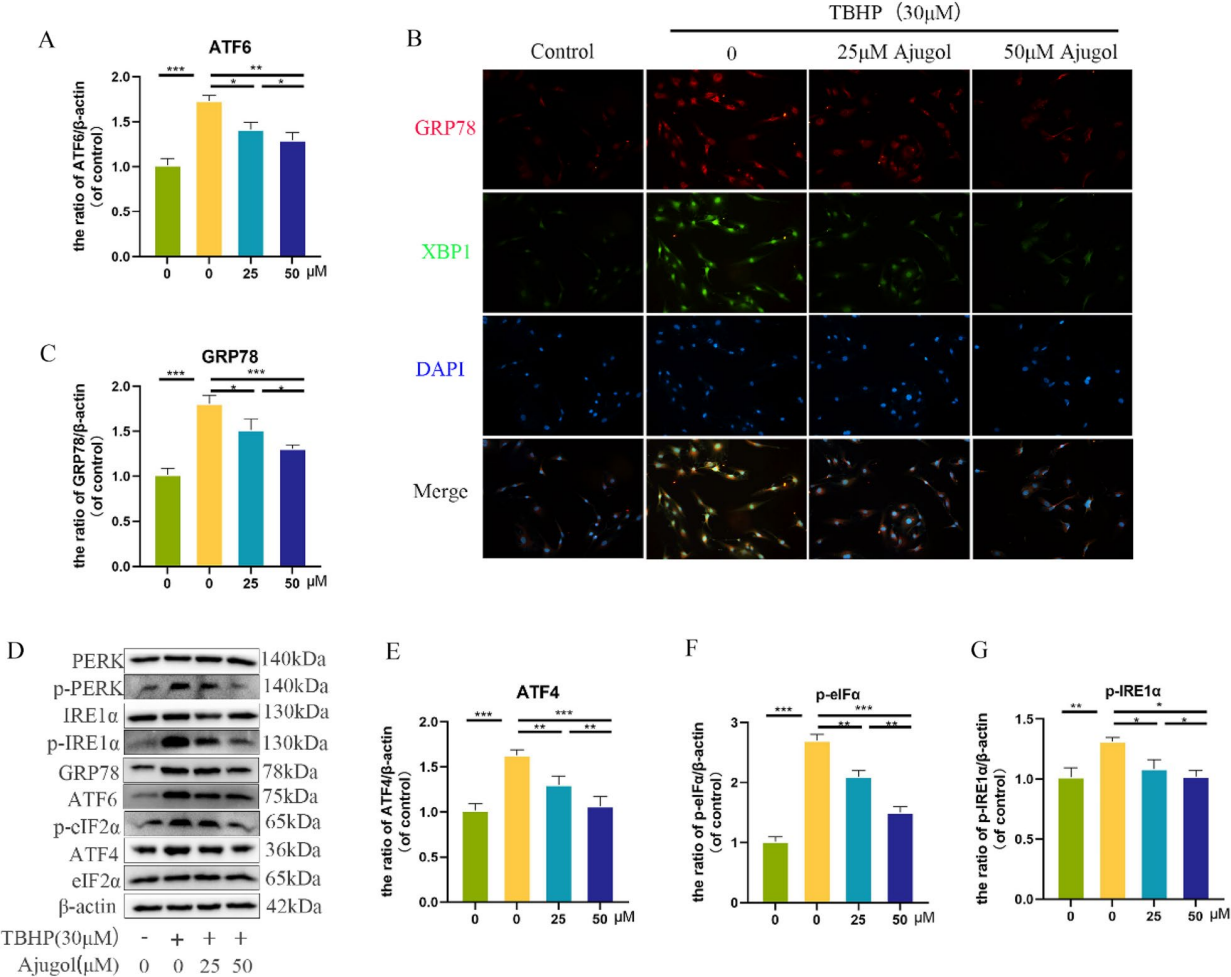
Ajugol alleviates TBHP-induced endoplasmic reticulum (ER) stress. **A**, **C**, **D**–**G** Protein levels of ER stress-related biomarkers, as detected and quantified by Western blot and ImageJ. **B** Double immunofluorescence staining of GRP78 and XBP1 was performed. All values are presented as mean ± standard deviation (n = 3). *P < 0.05,** *P* < 0.01,***P < 0.001

To investigate the mechanism by which Ajugol protects chondrocytes and attenuates TBHP-induced oxidative stress, we hypothesized that Ajugol may exert its protective effects by mitigating TBHP-induced endoplasmic reticulum (ER) stress. We performed Western blotting (WB) and immunofluorescence staining to assess key proteins involved in three representative pathways of ER stress: PERK-ATF4-eIF2α, ATF6, and IRE-XBP1. The results demonstrated that Ajugol at various concentrations significantly inhibited TBHP-induced ER stress, as evidenced by the expression levels of representative proteins examined via WB (Fig. 3A, C, D–G). Furthermore, compared to the other two pathways, it appears that PERK-ATF4-eIF2α undergoes more pronounced changes in response to Ajugol regulation. Similarly, the immunofluorescence experiments yielded similar results (Fig. 3B). These experimental findings collectively indicate that Ajugol can alleviate TBHP-induced endoplasmic reticulum stress.

### Ajugol activates TFEB-mediated autophagy

**Fig. 4:**
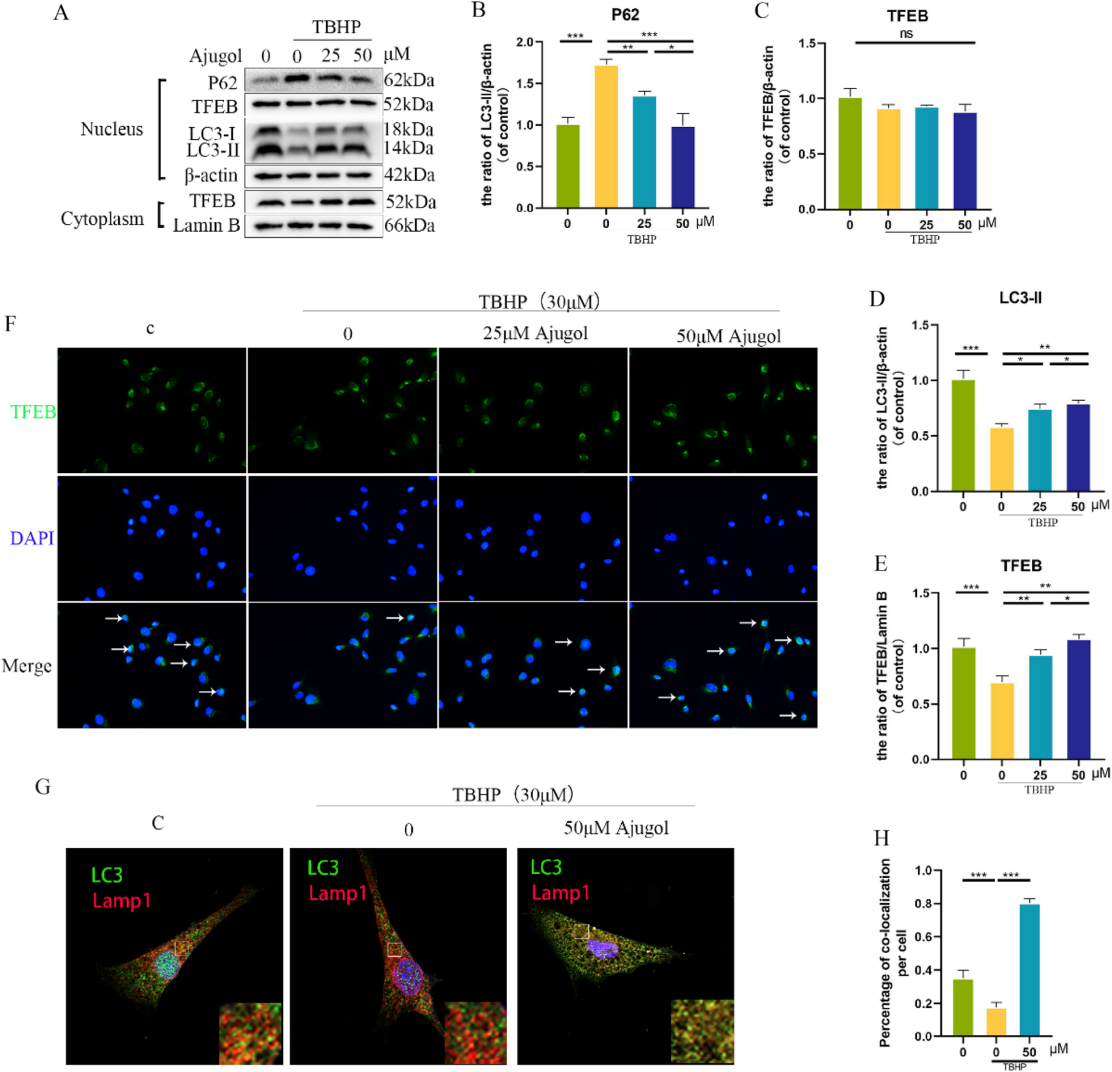
Ajugol activates TFEB-mediated autophagy. **A**–**F** Nuclear and cytoplasmic expression of P62, TFEB and LC-3. **G**, **H** LC3 and Lamp1 were co-localized by immunofluorescence All values are presented as mean ± standard deviation (n = 3). *P < 0.05,** *P* < 0.01,***P < 0.001

To further explore the effects of Ajugol in TBHP-stimulated chondrocytes, we hypothesized that it may activate TFEB-mediated autophagy. In the initial phase of our investigation, we employed molecular docking techniques to simulate the docking interaction between the TFEB protein and Ajugol, as depicted in Additional file 1: Figure S3. To validate this hypothesis, we initially assessed the differential expression of TFEB in the nucleus and cytoplasm using Western blotting (WB) and immunofluorescence staining. The results demonstrated that Ajugol increased the nuclear levels of TFEB in TBHP-stimulated chondrocytes (Fig. 4F). Activation of TFEB can induce cellular autophagy. To investigate the impact of Ajugol-induced TFEB activation on autophagy, we examined the levels of autophagy marker proteins, LC3 and P62, through WB (Fig. 4A–E). The results showed that Ajugol-induced TFEB activation successfully enhanced autophagy levels in chondrocytes. Additionally, we performed immunofluorescence double staining for LC3 and Lamp1. An increase in the yellow fluorescence area indicates enhanced autophagy within chondrocytes, further supporting our hypothesis (Fig. 4G).

### Silencing of TFEB reverses the ability of Ajugol to alleviate TBHP-induced endoplasmic reticulum stress

**Fig. 5:**
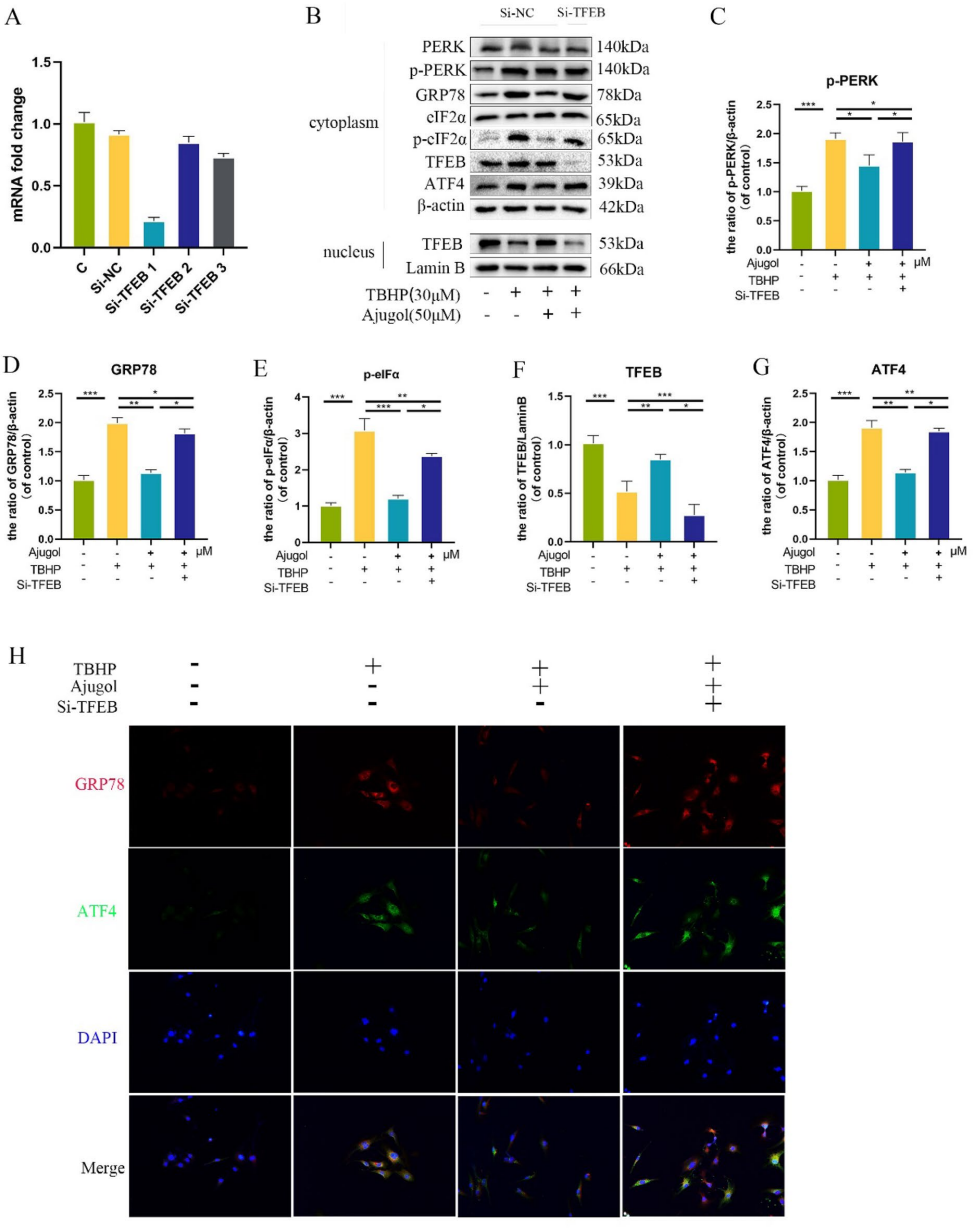
Silencing of TFEB reverses the ability of Ajugol to alleviate TBHP-induced endoplasmic reticulum stress. **A**TFEB mRNA expression after addition of si-TFEB. **B**–**G** The expression of PERK-eIF-ATF4 axis related proteins, and the expression of TFEB in the nucleus and cytoplasm. **H** Immunofluorescence of GRP78, ATF4 in chondrocytes. All values are presented as mean ± standard deviation (n = 3). *P < 0.05,** *P* < 0.01,***P < 0.001

To confirm whether Ajugol alleviates endoplasmic reticulum stress in chondrocytes through the activation of TFEB-mediated autophagy, we employed siRNA to knock down TFEB expression. First, we assessed the transfection efficiency using RT-PCR and observed a significant decrease in TFEB expression in cells transfected with TFEB siRNA (Fig. 5A). In previous experiments, we found that Ajugol may alleviate endoplasmic reticulum stress by inhibiting the PERK-eIF2α-ATF4 pathway. Thus, we performed Western blotting to evaluate the expression of representative proteins involved in this pathway and discovered that the protective effect of Ajugol on the PERK-eIF2α-ATF4 pathway was abolished upon TFEB silencing (Fig. 5B–G). Immunofluorescence staining yielded similar results (Fig. 5H). These findings indicate that silencing TFEB eliminates the ability of Ajugol to alleviate endoplasmic reticulum stress and protect chondrocytes.

### Silencing of TFEB reverses the anti-apoptotic and anti-ECM degradation effects of Ajugol on chondrocytes

**Fig. 6:**
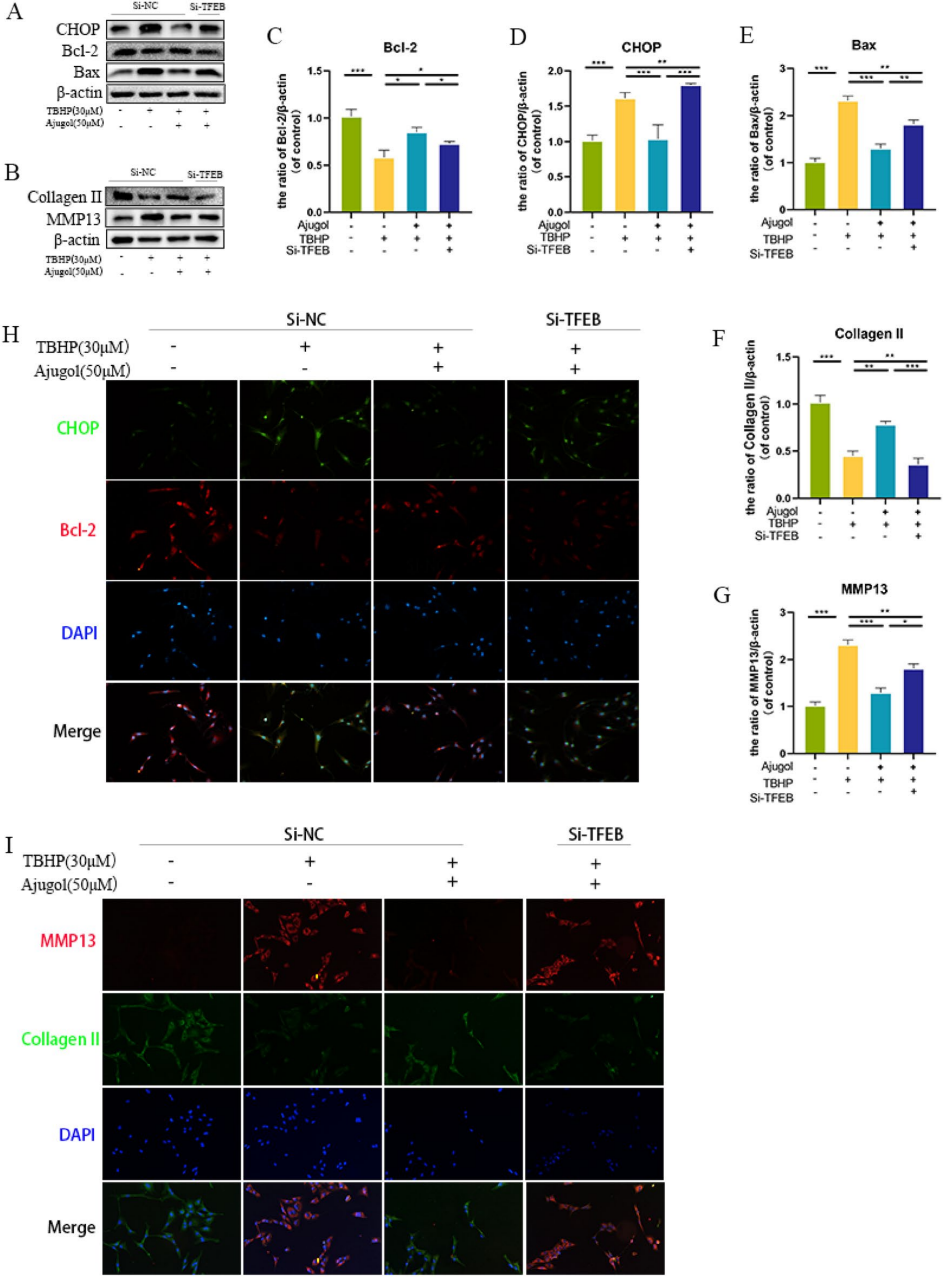
Silencing of TFEB reverses the anti-apoptotic and anti-ECM degradation effects of Ajugol on chondrocytes. **A**, **C**–**E** Protein expression of CHOP, Bcl-2 and Bax in chondrocytes. **B**, **F**, **G** Protein expression of Collagen II and MMP13 in chondrocytes. **H** Immunofluorescence of CHOP and Bcl-2 in chondrocytes. **I** Immunofluorescence of MMP13 and Collagen II in chondrocytes. All values are presented as mean ± standard deviation (n = 3). *P < 0.05,** P < 0.01,***P < 0.001

To further investigate whether silencing TFEB can reverse the anti-apoptotic effects of Ajugol, we performed WB experiments to validate our hypothesis. As shown in (Fig. 6A, C–E), silencing TFEB resulted in increased levels of proteins representing CHOP and Bax, while decreasing the level of Bcl-2. This indicates that siRNA-mediated TFEB knockdown can reverse the anti-apoptotic effects of Ajugol. Immunofluorescence staining showed an increase in CHOP-positive cells and a decrease in Bcl-2-positive cells, further supporting our findings (Fig. 6H). Next, we explored whether silencing TFEB could reverse the protective effect of Ajugol on ECM. WB and immunofluorescence staining revealed that silencing TFEB significantly reduced the level of Collagen II and increased the expression of MMP13, an enzyme involved in extracellular matrix degradation (Fig. 6B, F, G, I). To examine the overall ECM status of chondrocytes, Toluidine staining was performed on the cells. The results showed that silencing TFEB reversed the protective effect of Ajugol on the ECM (Additional file 1: Figure S1B). These results suggest that silencing TFEB can reverse the anti-apoptotic and anti-ECM degradation functions of Ajugol.

### CQ reverses Ajugol-induced protective effects in chondrocytes under oxidative stress

In the aforementioned experiments, we have demonstrated that Ajugol activates TFEB-mediated autophagy and mitigates cell death induced by ER stress. However, it remains unclear whether autophagy is the principal modulator of Ajugol's effects on ER stress. To further elucidate the underlying mechanisms, we employed the autophagy inhibitor chloroquine (CQ) to assess whether Ajugol's attenuation of ER stress is contingent upon the induction of autophagy. As shown in the Additional file 1: Figure S4A-H, the alleviating effect of Ajugol on TBHP-induced endoplasmic reticulum stress is partially reversed by CQ. Immunofluorescence staining results are consistent with the above (Additional file 1: Figure S4I–K). These findings suggest that Ajugol mitigates endoplasmic reticulum stress under oxidative stress conditions through the autophagy pathway in nature.

### Ajugol activates TFEB in vivo and improves the progression of osteoarthritis following DMM surgery

**Fig. 7:**
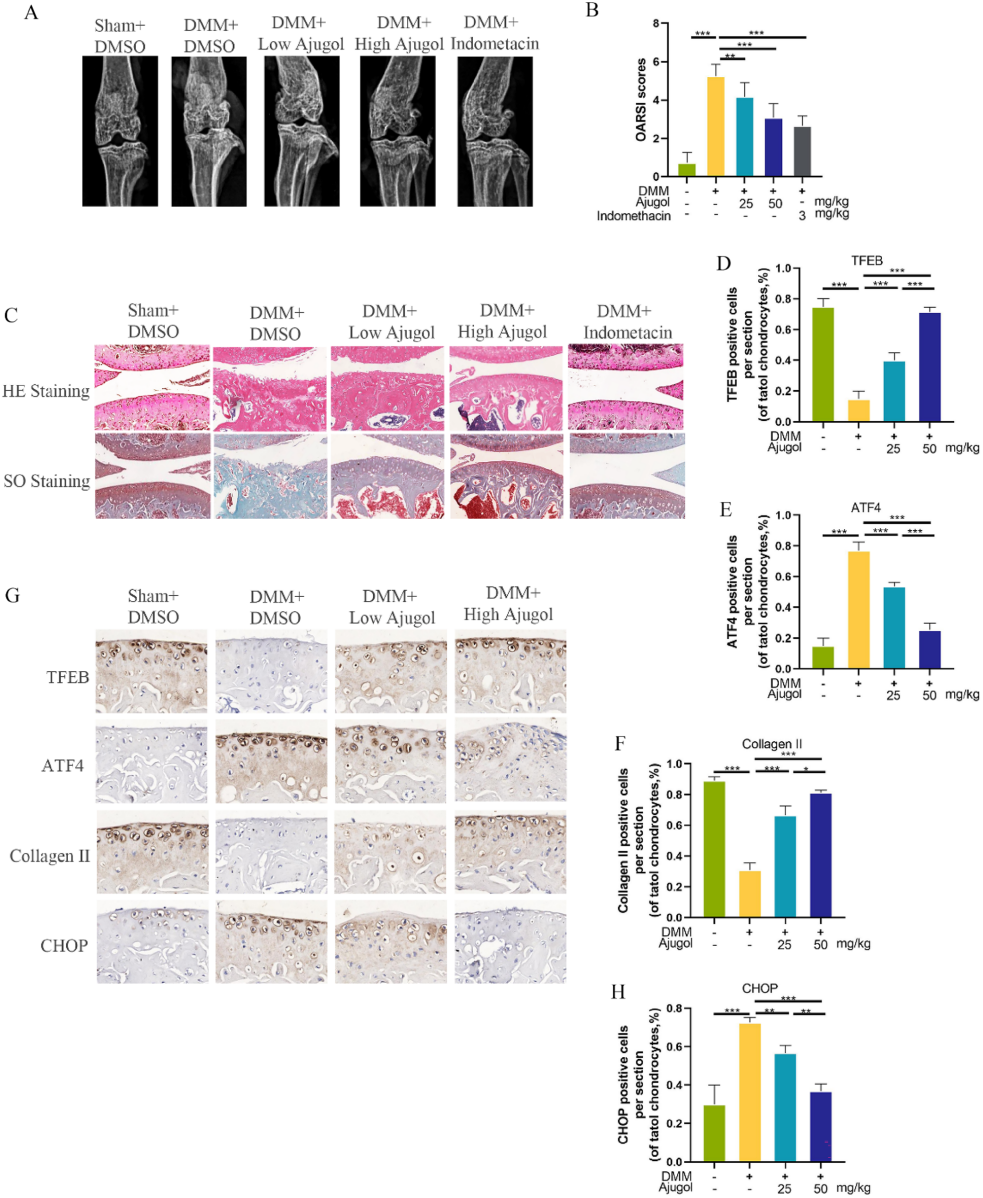
Ajugol activates TFEB in vivo and improves the progression of osteoarthritis following DMM surgery. **A** X ray of each group. **B** The severity of OA of different treatment groups based on OARIS scores. **C** Cartilage images after Safranin-O staining and HE staining for the effects of tangeretin on OA. **D**–**H** Immunohistochemical staining analysis for the expression of TFEB,ATF4,Collagen II and CHOP in the cartilage tissues

Subsequently, the therapeutic effects of Ajugol were evaluated in an in vivo mouse model of osteoarthritis (OA). The experimental design, administration protocol, and Ajugol concentrations were previously described. To facilitate a comprehensive assessment of Ajugol's potential in mitigating OA, we included the classical anti-inflammatory agent indomethacin as a comparative reference. Radiographic examination revealed cartilage sclerosis and joint space narrowing in the DMM (destabilization of the medial meniscus) group compared to the sham group. However, mice receiving graded intraperitoneal injections of Ajugol or indomethacin exhibited partial amelioration of OA progression (Fig. 7A). Subsequent Safranin O and H&E staining of tissue sections provided further insights. The DMM group exhibited marked cartilage defects and extensive proteoglycan loss, while those treated with Ajugol and indomethacin showed milder cartilage degradation in the knee joint tissue. Moreover, Osteoarthritis Research Society International (OARSI) scoring was employed to gauge the severity of OA in each group. Results revealed the highest scores in the DMM group, whereas the DMM + Ajugol and DMM + indomethacin groups exhibited a partial reversal of this trend (Fig. 7B). Finally, immunohistochemical analysis was performed on tissue sections from all mice to investigate alterations in TFEB, ATF4, Collagen II, and CHOP expression within the OA model. Notably, TFEB and Collagen II were found to be reduced, while CHOP and ATF4 were increased in the OA group (Fig. 7D–H). Remarkably, administration of Ajugol and indomethacin effectively counteracted these changes. In conclusion, Ajugol demonstrates promising efficacy in attenuating OA progression in the DMM mouse model.

## Discussion

Worldwide, it is estimated that 240 million people suffer from symptomatic and functionally impaired osteoarthritis (OA). The knee joint is the most commonly affected joint, with nearly 30% of individuals aged 45 and above having radiographic evidence of knee OA, and approximately half of them experiencing knee joint symptoms [33, 34]. OA imposes a significant economic burden and is associated with increased mortality. Although OA itself is not life-threatening, individuals with OA often have comorbidities that contribute to a roughly 20% higher mortality rate [35]. Indeed, the management of OA typically involves a multi-faceted approach that includes education, exercise, weight management, and the use of medications such as nonsteroidal anti-inflammatory drugs (NSAIDs) and corticosteroids [36–39]. These strategies aim to alleviate pain, reduce inflammation, improve joint function, and slow down the progression of the disease. However, it is true that there is currently no definitive and rapid-acting pharmacological treatment for OA. Therefore, finding effective pharmacological interventions for the control of OA is of paramount importance.

In recent years, advancements in molecular biology research have led to the identification of an increasing number of molecular targets and signaling pathways that are implicated in the onset and progression of OA. These discoveries have provided valuable insights into the underlying mechanisms of the disease. The endoplasmic reticulum (ER), the largest organelle in the cell, plays a crucial role in protein synthesis, folding, and maintaining cellular homeostasis [40]. Under conditions such as nutrient deprivation, hypoxia, oxidative stress, calcium imbalance, viral infection, or the accumulation of misfolded or unfolded proteins, the ER becomes overwhelmed and unable to properly fold and process proteins, resulting in ER stress [41, 42]. Concurrently, the cell initiates the unfolded protein response (UPR) to alleviate the burden on the ER. The UPR primarily involves three pathways: PERK, ATF6, and IRE1 [43, 44]. These pathways coordinate processes such as autophagy, ER-phagy, and ER-associated protein degradation (ERAD) to alleviate ER stress and restore cellular homeostasis [45, 46]. However, when ER stress surpasses the cell's capacity, it can lead to apoptosis and trigger a cascade of pathological processes.

The preceding discussion highlighted the role of proper autophagy in maintaining ER homeostasis. Autophagy, an intracellular process involving the degradation and recycling of cellular components, such as damaged organelles, misfolded proteins, and other cellular debris, is key to this mechanism [47, 48]. TFEB controls the expression of multiple genes associated with autophagy, lysosomal biogenesis, and lysosomal function [49, 50]. Under normal circumstances, TFEB resides in the cytoplasm, phosphorylated and thus inactive. However, during cellular stress or nutrient deprivation, TFEB undergoes dephosphorylation and translocates to the nucleus, where it binds to promoter regions of target genes involved in autophagy and lysosomal function [51]. Our research focuses on pharmacologically activating TFEB to induce its phosphorylation, thereby promoting autophagy and subsequently alleviating ER stress.

**Fig. 8:**
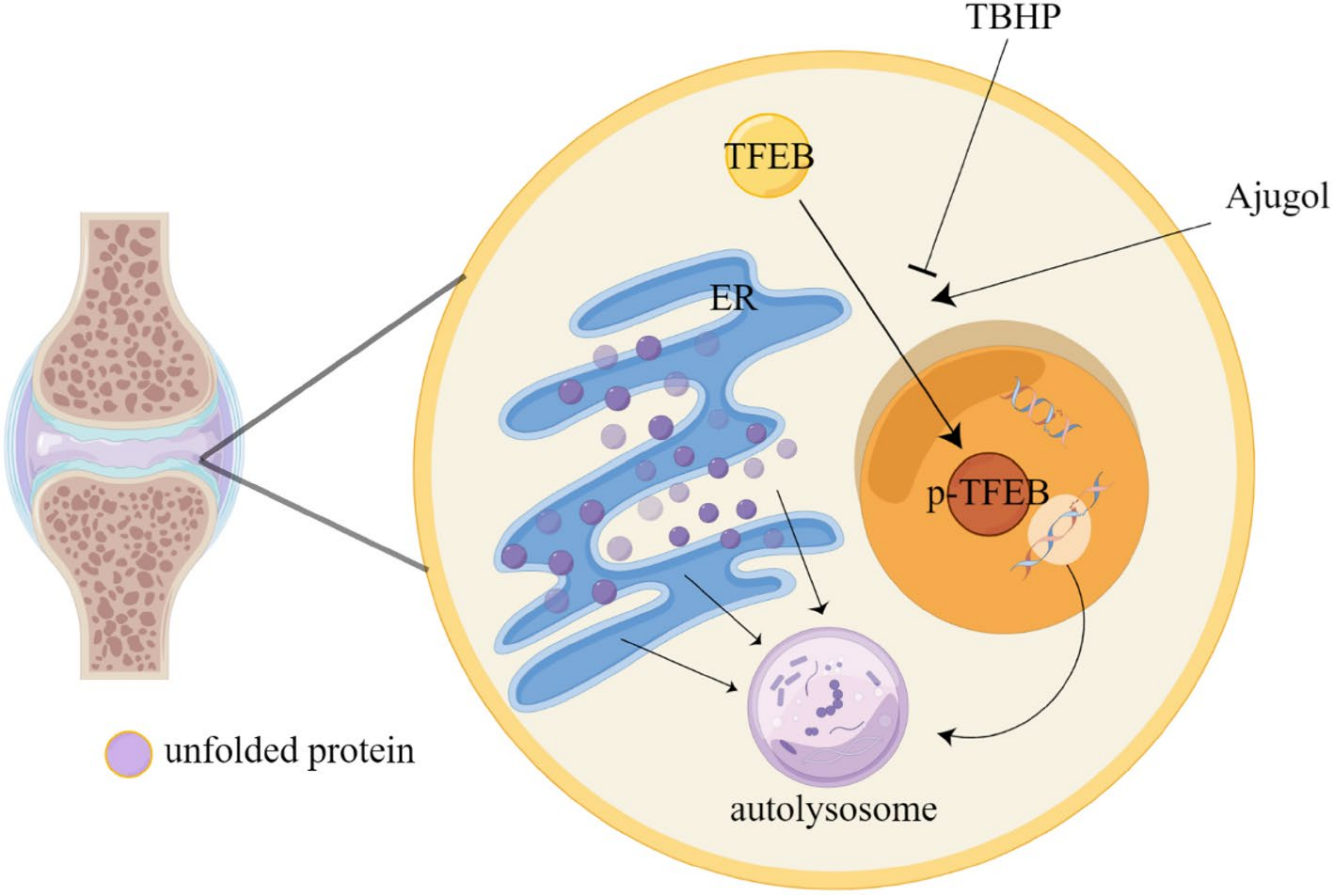
Role of Ajugol in osteoarthritis. Ajugol alleviates oxidative stress-induced endoplasmic reticulum stress and ameliorates the progression of OA by promoting nuclear translocation of TFEB

Rehmannia glutinosa, a perennial herbaceous plant widely distributed in China and East Asia, is of significant medicinal value with its root being the primary part used in traditional Chinese medicine [52]. It is believed to possess hepatorenal tonic, qi and blood replenishing, heat-clearing, and blood-cooling effects. However, the specific constituents in Rehmannia glutinosa responsible for treating diseases are not yet fully understood. One such constituent, Ajugol, is an extract derived from Rehmannia glutinosa. Recent studies have shed light on its effects on the digestive, respiratory, and nervous systems. However, its potential role in the musculoskeletal system, such as osteoarthritis, remains unclear. Therefore, for the first time, we introduced Ajugol into OA (osteoarthritis) research and discovered its ability to activate TFEB-mediated autophagy, thereby alleviating ER stress (Fig. 8). In both in vivo and in vitro experiments, it was observed that Ajugol mitigated chondrocyte apoptosis and alleviated the progression of osteoarthritis. Notably, its efficacy showed no significant difference compared to the classical anti-inflammatory drug indomethacin.

In our experiments, we have demonstrated that Ajugol can regulate TFEB. In previous studies, various pathways such as the AMPK pathway and mTOR pathway have been shown to regulate TFEB downstream [53, 54]. However, we were unable to determine whether Ajugol directly regulates TFEB or indirectly modulates TFEB through the regulation of AMPK or mTOR. Furthermore, we have shown that Ajugol can activate TFEB and alleviate ER stress. However, it remains uncertain whether Ajugol exclusively relies on TFEB to regulate autophagy and alleviate ER stress or if there are other mechanisms involved. Further investigations are needed to elucidate the precise molecular mechanisms by which Ajugol exerts its effects on TFEB and autophagy regulation, as well as its role in mitigating ER stress. Furthermore, we were unable to stain the sections for clear hyaline cartilage (HC) and calcified cartilage (CC), which would have provided more favorable histological evidence [55, 56]. Understanding these mechanisms will provide valuable insights for potential therapeutic applications of Ajugol in the context of ER stress-related conditions.

### Supplementary Information

**Additional file 1: Figure S1.** (A, B):Chondrocytes Toluidine blue staining. **Figure S2.** ROS detection of Chondrocytes. **Figure S3.** Molecular docking map of TFEB and Ajugol. **Figure S4.** CQ reverses Ajugol-induced protective effects in chondrocytes under oxidative stress. (A-H) Western blot results of PERK, p-PERK, GRP78, eIF2α, p-eIF2α and ATF4, as well as their quantification bar charts.(I-K) GRP78 and immunofluorescence ATF4 diagram and their quantitative.

## Data Availability

The data that support the finding of this study are available from the corresponding author upon reasonable request.
