# Supplementary material for: Ajugol's upregulation of TFEB-mediated autophagy alleviates endoplasmic reticulum stress in chondrocytes and retards osteoarthritis progression in a mouse model
Source: Chin Med. 2023 Sep 7;18:113. doi: 10.1186/s13020-023-00824-7 (PMC10483732; doi:10.1186/s13020-023-00824-7)
Supplement: Supplementary file 1 — Additional file 1: Figure S1. (A, B):Chondrocytes Toluidine blue staining. Figure S2. ROS detection of Chondrocytes. Figure S3. Molecular docking map of TFEB and Ajugol. Figure S4. CQ reverses Ajugol-induced protective effects in chondrocytes under oxidative stress. (A-H) Western blot results of PERK, p-PERK, GRP78, eIF2α, p-eIF2α and ATF4, as well as their quantification bar charts.(I-K) GRP78 and immunofluorescence ATF4 diagram and their quantitative. [file 13020_2023_824_MOESM1_ESM.docx]

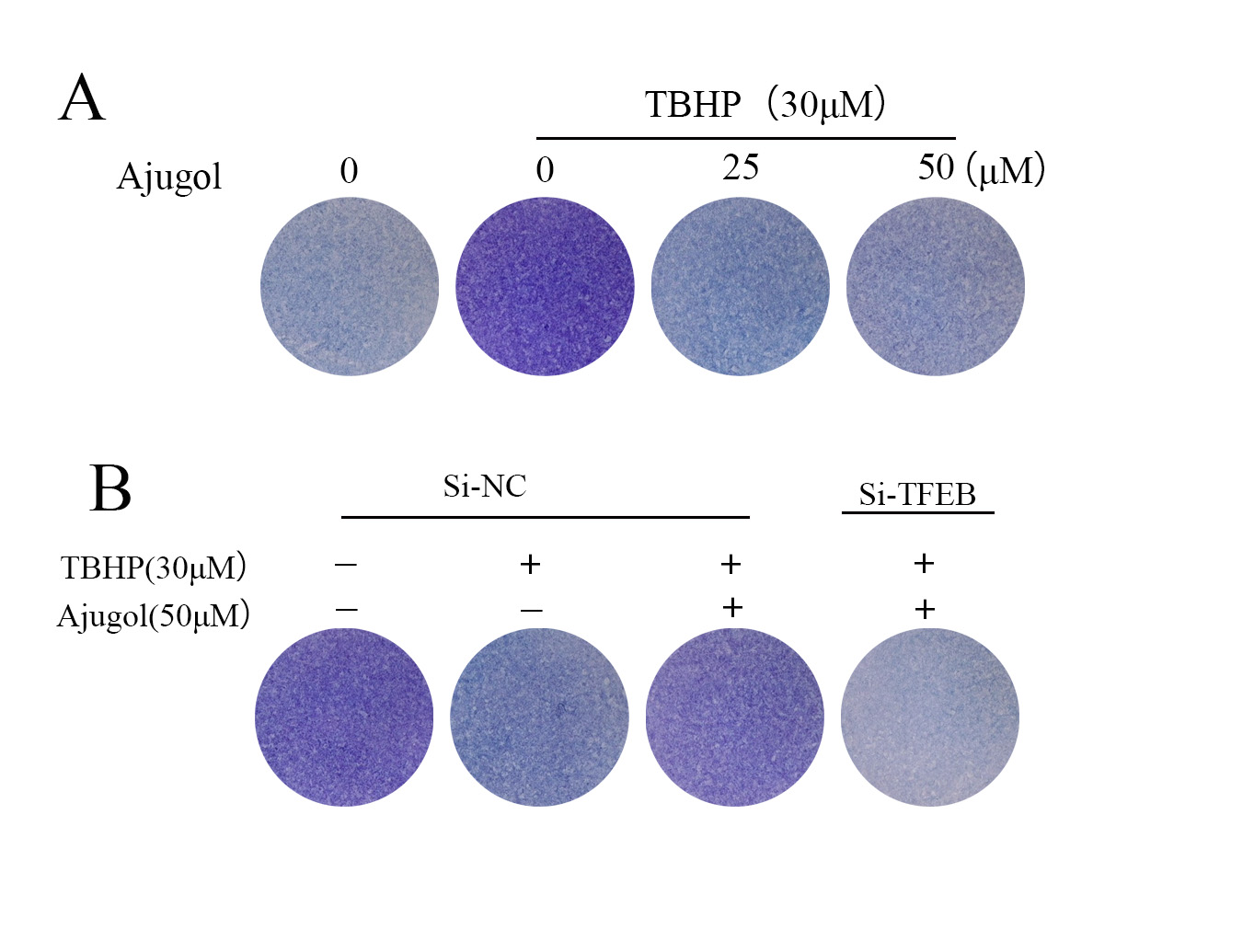


**Fig.S1:(A,B):Chondrocytes Toluidine blue staining.**
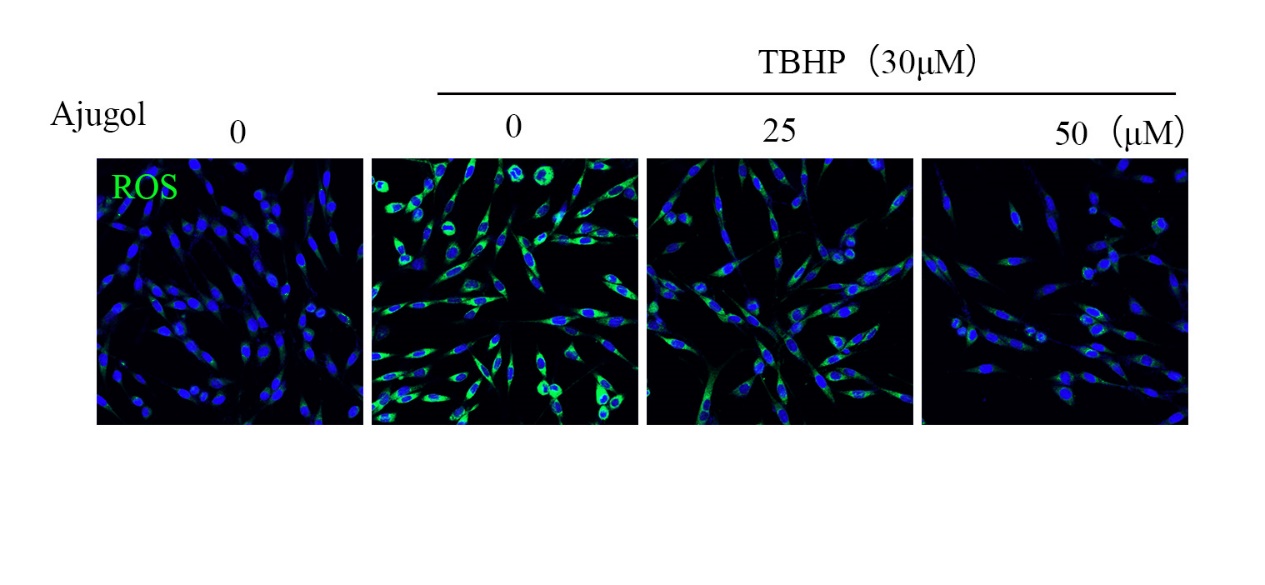


**Fig.S2: ROS detection of Chondrocytes.**


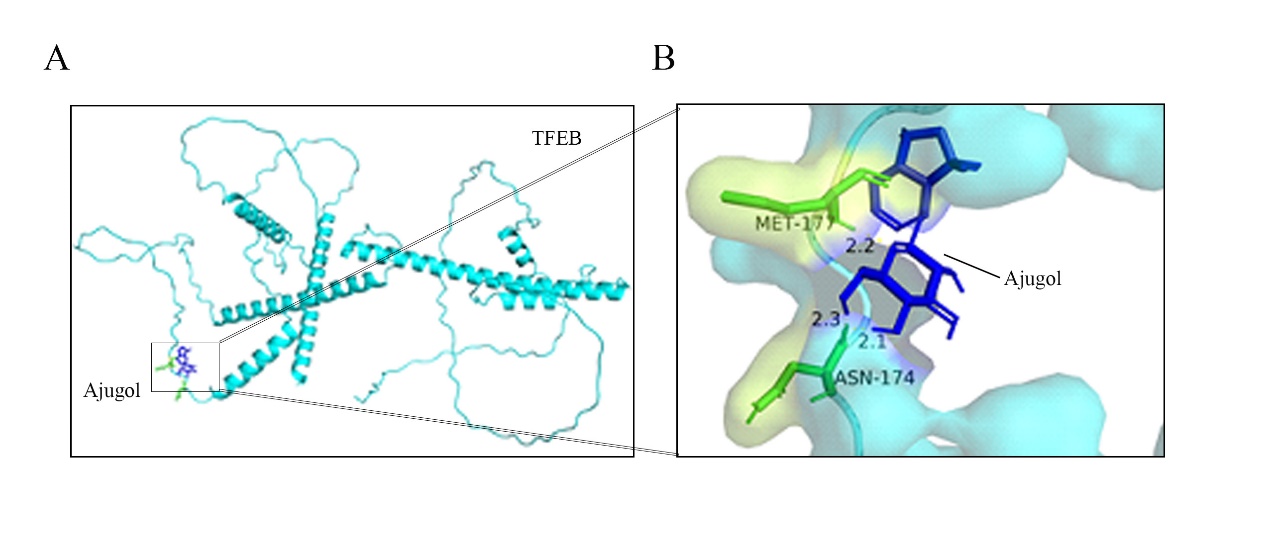


**Fig.S3: Molecular docking map of TFEB and Ajugol**


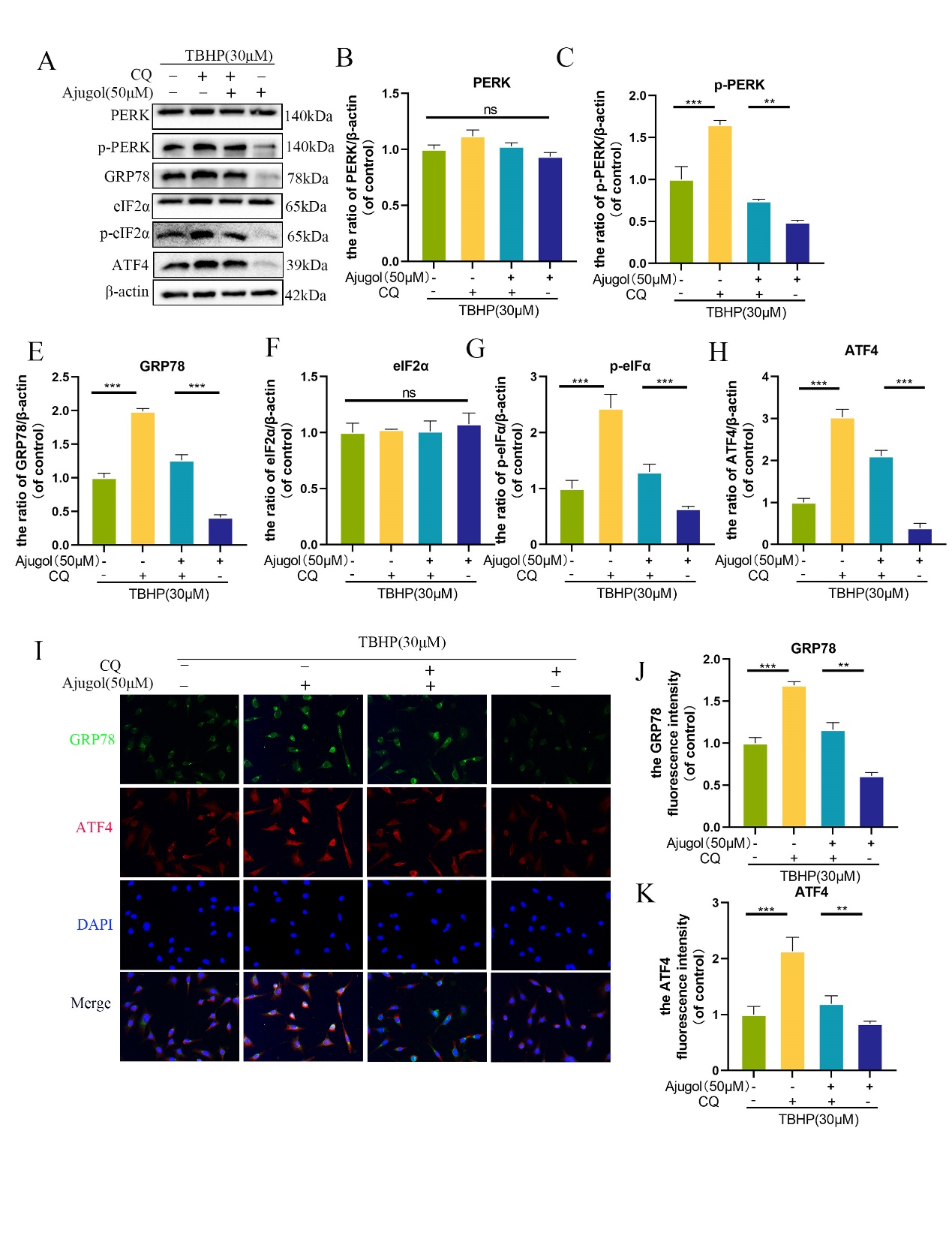


**Fig.S4: CQ reverses Ajugol-induced protective effects in chondrocytes under oxidative stress. (**A-H) Western blot results of PERK, p-PERK, GRP78, eIF2α, p-eIF2α and ATF4, as well as their quantification bar charts.(I-K) GRP78 and immunofluorescence ATF4 diagram and their quantitative.
